# Molecular Iodine Capture by Covalent Organic Frameworks

**DOI:** 10.3390/molecules27249045

**Published:** 2022-12-19

**Authors:** Yuting Yang, Changzheng Tu, Hongju Yin, Jianjun Liu, Feixiang Cheng, Feng Luo

**Affiliations:** 1 College of Chemistry and Environmental Science, Qujing Normal University, Qujing 655011, China; 2 School of Chemistry, Biology and Materials Science, East China University of Technology, Nanchang 330013, China

**Keywords:** covalent organic frameworks, mechanisms, electron-rich groups, charge transfer, iodine capture

## Abstract

The effective capture and storage of volatile molecular iodine from nuclear waste is of great significance. Covalent organic frameworks (COFs) are a class of extended crystalline porous polymers that possess unique architectures with high surface areas, long-range order, and permanent porosity. Substantial efforts have been devoted to the design and synthesis of COF materials for the capture of radioactive iodine. In this review, we first introduce research techniques for determining the mechanism of iodine capture by COF materials. Then, the influencing factors of iodine capture performance are classified, and the design principles and strategies for constructing COFs with potential for iodine capture are summarized on this basis. Finally, our personal insights on remaining challenges and future trends are outlined, in order to bring more inspiration to this hot topic of research.

## 1. Introduction

As one of the clean energy sources that is most likely to replace fossil fuels, nuclear energy plays an extremely important role in many countries [1,2]. However, the utilization of nuclear energy faces a major problem related to the safe disposal of nuclear waste containing radioactive substances, especially radioactive iodine, which is difficult to handle in the actual environment due to its volatility, strong fluidity, and fast diffusion [3,4]. The main radioisotopes for iodine are ^129^I and ^131^I. ^129^I is considered to be one of the most dangerous byproducts in nuclear waste due to its long radioactive half-life (1.57 × 10^7^ years) and negative effects on human metabolic processes (it can be accumulated in the human thyroid gland, causing serious diseases) and the environment. Additionally, although ^131^I has a short half-life (about 8 days), it is often combined with other hydrocarbons to yield organic compounds, such as methane iodide, which also make it extremely harmful to its ecological surroundings and human health [5,6,7]. If handled improperly, it will seriously restrict the development and application of nuclear energy. On the other hand, radioactive iodine has important applications in the medical field. For example, ^125^I seed implantation is widely applied in clinical brachytherapy, and ^131^I can be used for the examination of thyroid function and the treatment of malignant tumors [8]. In this regard, it is urgent to develop a highly efficient method to capture and store radioactive iodine.

It is known that the possible radioactive iodine species in the environment are iodate (IO_3_^−^), molecular iodine (I_2_), and organic iodine species (e.g., methyl iodide (CH_3_I) and ethyl iodide (CH_3_CH_2_I)). Different iodine species need to be handled in different ways [9,10,11]. Among them, volatile molecular iodine (I_2_) is the main chemical form of radioiodine in fission, which is of major concern due to its chemical and biological toxicities. Given this, developing functional adsorbents to efficiently capture radioiodine vapor is extremely significant. The traditional adsorbents developed for radioactive molecular iodine capture and storage are mainly inorganic adsorbents, such as zeolites, Ag-doped silica aerogels, clay, and activated carbon [12,13,14,15,16]. However, these inorganic adsorbents normally have low efficiency, high cost, instability toward moisture, or demanding application scenarios. For instance, the theoretical and practical adsorption capacities of Ag-doped silica aerogel are 1.18 g·g^−1^ and 0.10–0.31 g·g^−1^, respectively, and are far from meeting actual requirements [17]. For the elimination of radioiodine in complicated conditions, it is necessary to design novel materials with high sorption capacity, high stability, high selectivity, and low cost.

**Scheme 1:**
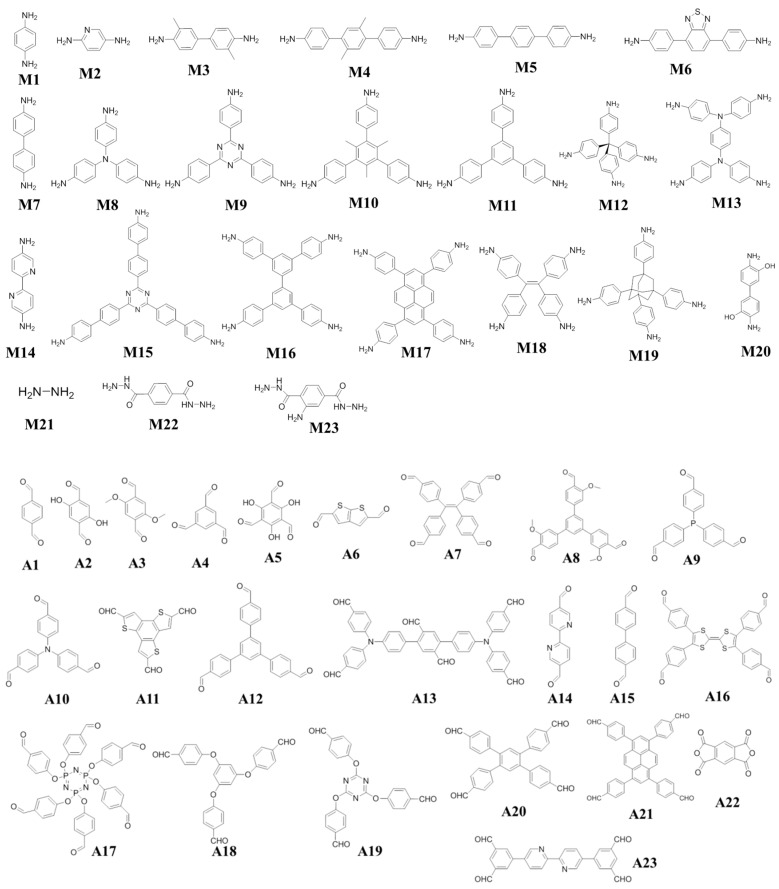
Various monomers and functional building blocks (amine groups and aldehyde groups) used for the construction of COFs with iodine capture performance. A—aldehyde; M—amine.

In recent years, the application of metal—organic frameworks (MOFs) and porous organic polymers (POPs) for iodine capture has attracted great interest. MOFs have been widely studied as adsorbents for molecular iodine because of their high surface area [18,19,20,21,22,23,24,25]. Yet, the stability of MOFs at high temperatures and in solution are generally poor, which limits their practical application. POPs, including hypercrosslinked polymers (HCPs) [26,27,28], conjugated microporous polymers (CMPs) [29,30,31,32], porous aromatic frameworks (PAFs) [33,34,35], and covalent organic frameworks (COFs) [36], are another type of porous material with higher stability that are connected via strong covalent bonds. They have been found to have a high potential for capturing and storing iodine, and many of them have achieved very high iodine capacities. As a unique class of POPs, COFs are distinguished from other POPs by their highly ordered internal structures and crystallinities, as well as their advantages of easy functionalization, low density, large BET surface area, intrinsic porosity, and superior chemical/thermal stability [36,37,38,39]. The fascinating features of COFs, with their atomically precise integration of scaffolds into 2D/3D topologies, have shown outstanding applications in many fields, such as gas adsorption, sensing, energy conversation, catalysis, etc. Since COF-1 and COF-5 were reported by Yaghi’s group [40] for the first time in 2005, COF materials have become a research focus of the current scientific and technological frontier. Several reviews have summarized the synthesis, characterization, and application of COFs [41,42,43,44,45,46]. However, there is still lack of reviews systematically focusing on the COFs applied in the iodine capture area. In 2017, Zhao et al. first applied COFs in the field of iodine capture and achieved remarkable results [47]. They reported a heteropore 2D COF (SIOC-COF-7), which showed an I_2_ adsorption capacity of 4.81 g·g^−1^ due to its large inner cavities, and a special structure of porous shells. This 2D COF also showed good adsorption performance towards dissolved I_2_ in the solution phase. Since then, many successful examples of COFs constructed by various monomers and functional building blocks (Scheme 1) have been reported and applied in the iodine capture area in recent years. These COFs, with their specific pore environments and tunable chemistry, can be easily functionalized to acquire effective iodine capture active sites. Although research on the application of COFs in iodine capture is still in its infancy, the unique structural characteristics of these materials, such as their tunable pore size, large surface area, and high crystallinity, make them highly competitive candidates for iodine capture applications.

This review intends to concisely summarize recent progress in research on iodine adsorption by COFs. The purpose is to discuss the design and synthesis strategies of COFs that are useful for iodine capture. Additionally, the drawbacks of COFs in iodine capture will be discussed; for example, most of them were evaluated at relatively low temperatures (about 75 °C) and at high I_2_ concentrations (>10,000 ppmv).

## 2. Mechanism of Iodine Capture by COFs

### 2.1. Methods for Studying Adsorption Mechanism

The mechanism of the iodine capture process by COFs mainly consists of physical adsorption, chemical adsorption, and a combination of physical and chemical adsorption. Detailed research on the adsorption mechanism could deepen our understanding of the adsorption process, and thus, promote the theoretical development of the design of COF-based materials with iodine-adsorption properties. In order to explore the adsorption mechanism, samples of COFs loaded with iodine (expressed as I_2_@COF) are usually studied via FT-IR spectra, Raman spectra, X-ray photoelectron spectroscopy (XPS), electron paramagnetic resonance (EPR), PXRD patterns, transmission electron microscopy (TEM), density functional theory (DFT) calculations, etc. The results of all analyses should be consistent with each other.

**Figure 1:**
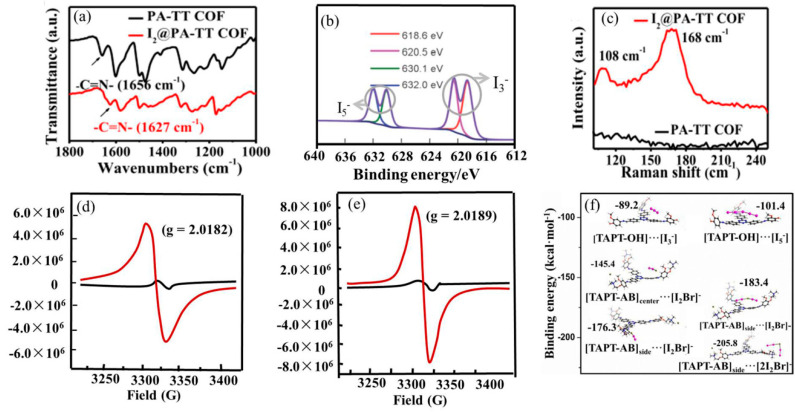
(**a**) FT-IR spectra of PA-TT COF and I_2_ @PA-TT COF. Reprinted/adapted with permission from Ref. [48]. Copyright (2023), Elsevier. (**b**) Iodine high-resolution XPS spectra of I_2_ @TTF-TD-COF. Reprinted/adapted with permission from Ref. [49]. Copyright (2022), Springer-Verlag GmbH. (**c**) Raman spectra of PA-TT COF and I_2_ @PA-TTCOF. Reprinted/adapted with permission from Ref. [48]. Copyright (2023), Elsevier. EPR spectra of JUC-560 (**d**) and JUC-561 (**e**) before (black curve) and after (red curve) iodine uptake. Reprinted/adapted with permission from Ref. [50]. Copyright (2021), Royal Society of Chemistry. (**f**) Density functional theory (DFT) calculations of the binding energies of TAPT-OH with I_3_^−^ and I_5_^−^, and TAPT-AB with [I_2_Br]^−^ and [2I_2_Br]^−^ located at different positions. Reprinted with permission from Ref. [51]. Copyright (2021), John Wiley and Sons.

The fT-IR spectra of the original samples and those after the loading of iodine were compared. If the characteristic peak positions shift obviously or gradually decrease, or even disappear, it can be proven that there is chemical adsorption caused by the charge-transfer interaction between iodine and electron-rich groups. It can also enable us to infer which structural parts of COFs are related to the adsorption of I_2_. For example, as shown in Figure 1a, compared with the FT-IR spectra of PA-TT COF, the stretching vibration peak of the -C=N- of I_2_@PA-TT COF shows a significant shift from 1656 cm^−1^ to 1627 cm^−1^, indicating the existence of a chemisorption process caused by the charge-transfer interaction between iodine and the N atom of C=N [48].

X-ray photoelectron spectroscopy (XPS) was conducted to investigate the existing state of iodine captured in COFs (such as I_3_^−^ and I_5_^−^). Before iodine adsorption, the COFs exhibit no obvious peaks. After adsorption, strong peaks appear in the range of 617~620 eV and 629~632 eV for I_3_^−^ and I_5_^−^, respectively (Figure 1b). So, it can be deduced that the absorbed iodine species in COFs existed as polyiodide anions.

Raman spectra were also used to reveal the chemical state of iodine inside the pores of COFs. No obvious peaks are found in COFs before the uptake of iodine. The intense peaks at around 107~109 cm^−1^ and 167~170 cm^−1^ emerge for I_2_@COFs, which can be attributed to the I_3_^−^ and I_5_^−^ ions, respectively (Figure 1c). If the peaks of I_2_, I_3_^−^, and I_5_^−^ can be observed simultaneously, it indicates that elemental iodine and polyiodide anions co-existed in the channels of COFs and the iodine adsorption process was a combination of physisorption and chemisorption. XPS and Raman spectroscopy are the two most effective methods for revealing the chemical state of iodine within the pores of COFs.

The generation of radical cations after iodine adsorption was confirmed via electron paramagnetic resonance (EPR) studies. The original samples show a very weak EPR signal, while there is an approximate increase of two orders of magnitude in paramagnetic intensity after I_2_ doping. For example, the EPR studies on JUC-560 and JUC-561 show obvious peaks at *g* = 2.0182 and 2.0189, respectively (Figure 1d,e), clearly indicating the presence of TTF·^+^ radical cations oxidized by iodine [50].

Density functional theory (DFT) calculations can help researchers gain insight into how the COF frameworks bind iodine. It is well known that the binding energy can remarkably affect iodine capture. Normally, the stronger the interaction between iodine and the adsorption sites, the higher the iodine uptake. The binding energy between the model compounds was calculated via DFT and the corresponding conclusions were obtained by comparing the values. Han’s group [51] calculated the binding energies of the model molecules TAPT-OH and TAPT-AB with the identified iodine species. As shown in Figure 1f, the binding energies between TAPT-AB and [I_2_Br]^−^/[2I_2_Br]^−^ are approximately twice that between TAPT-OH and I_3_^−^/I_5_^−^, suggesting a stronger affinity of TAPT-AB toward the iodine species. This is consistent with expectations and explains the experimental observations.

PXRD and transmission electron microscopy (TEM) analysis can show whether the crystallinity and morphology of COFs can be maintained throughout the iodine capture process.

### 2.2. Physical Adsorption of Iodine by COFs

Physical adsorption mainly depends on the surface areas, pore sizes and pore volumes of COFs. Thus, the functionalized architectures are designed to have high specific surface areas, and large pore sizes and pore volumes for efficient physisorption of iodine. For example, Liu et al. designed and synthesized four 2D COFs with different pore sizes for volatile iodine adsorption [52]. Finally, they revealed that the iodine uptake capacity was not only determined by the pore volume but also significantly affected by the intrinsic pore size.

Jiang’ group reported a series of 2D COFs with 1D open channels, which possessed various topologies from hexagonal to tetragonal and trigonal, and were free of specific binding sites and interchannel interpenetration [53]. So, the possibility of charge transfer was excluded. Among these 2D COFs, a TPB-DMTP-COF with a pore size of 3.3 nm and a pore volume of 1.28 cm^3^·g^−1^ achieved a remarkable iodine adsorption capacity (6.26 g·g^−1^) only in physical adsorption, which was driven by van der Waals forces. They proved that I_2_ capture does not require specific functionalization of the porous skeleton, and that pores of any shape or size can be 100% occupied by physical adsorption. However, its adsorption kinetics were quite slow (0.13 g·g^−1^·h^−1^).

### 2.3. Synergistic Effect of Physical and Chemical Adsorption

As aforementioned, porosity plays an important role in the adsorption of iodine vapor. However, in addition to the amount of adsorption, the adsorption rate is also a significant factor that must be considered. As for the TPB-DMTP-COF, it has an ultrahigh uptake capacity but takes 100 h to reach saturation, which is particularly unfavorable in the case of emergency disasters. Studying only physical adsorption—whereby the iodine uptake capacity of COF materials is determined by pore volume, while the iodine uptake kinetics are determined by pore connectivity and size—limits the application of COFs for iodine capture. The combination of physical and chemical adsorption is of paramount importance in the designed synthesis of COF materials for the capture of radioiodine. It is commonly believed that the interaction of I_2_ with electron-rich groups (that is, the so-called active sites, such as C=N, -NH_2_, triazine, pyridine, aromatic rings, etc., which can effectively adsorb electron-deficient I_2_ via the formation of charge-transfer complexes) and pore channels results in a combination of physical and chemical adsorption for the I_2_ capture process. The iodine vapor adsorbed into the pores through physical adsorption can readily generate the polyiodide species I_3_^−^ and I_5_^−^ due to the strong interactions between the exposed electron-rich groups and I_2_, implying that a chemisorption process occurs. In the meantime, iodine molecules may fill the remaining pore channels through physical adsorption.

**Figure 2:**
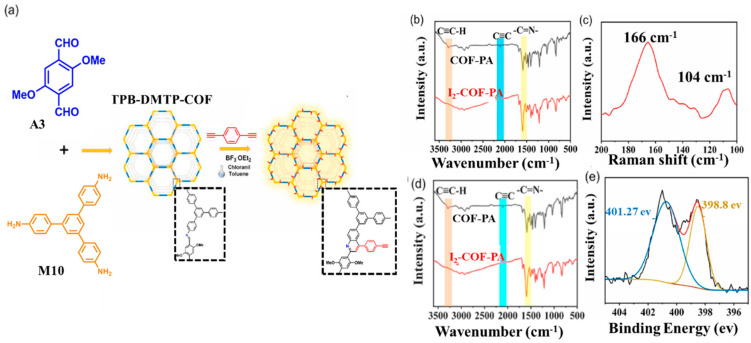
(**a**) Synthetic route. (**b**) FT-IR spectra. (**c**) Raman spectra of I2@COF-PA. (**d**) FT-IR spectra of COF-PA and I_2_@COF-PA. (**e**) XPS survey spectrum. Reprinted/adapted with permission from Ref. [54]. Copyright (2021), Elsevier.

At present, most COFs for iodine capture are based on the above principles. Zhao and co-workers prepared a 2D COF (COF-PA) containing quinoline and phenylacetylene units via post-functionalization of the TPB-DMTP-COF (A3-M10, Figure 2) [54]. XPS and Raman spectra prove that electrons are transferred from electron-rich quinoline units to electron-deficient iodine. They can not only form complex electron-deficient I_2_ via an electron-rich quinoline unit, but can also adsorb I_2_ via a chemical reaction with phenylacetylene moieties. Although the surface area and pore size of COF-PA were reduced compared with before functionalization, the adsorption rate was accelerated and the adsorption capacity was still high at low iodine concentrations. Very recently, Zhai et al. discovered two rare cationic COFs, C-TP-PDA-COFs and C-TP-BPDA-COFs (A5-M2, A5-M13), via a post-function process. The cationic C-TP-BPDA-COF exhibits a higher iodine capture value (6.11 g·g^−1^) than that of neutral COF [55].

The mechanism study found that after the adsorption of iodine by COF, before ionization, only the peak of the neutral iodine molecule existed in the XPS spectra, which suggests typical physical adsorption. However, after ionization, signals for both neutral I_2_ and anion I_3_^−^ signals were observed. This indicates the presence of both physical and chemical adsorption. Chang et.al reported that two tetrathiafulvalene (TTF)-based COFs, JUC-560 (A16-M4) and JUC-561(A16-M8), achieve excellent iodine adsorption capacity (8.19 g·g^−1^) and ultrafast adsorption kinetics (0.70 g·g^−1^·h^−1^) [50]. These architectures are designed to have large specific surface areas for high iodine uptake through the physical process, and plentiful TTF functional groups for chemisorption. The synergistic effects of the physisorption and chemisorption processes together contributed to the superior iodine vapor adsorption capacities of the studied COF materials.

The above description indicates that the integration of the virtues of physical and chemical adsorption is of paramount importance in the designed synthesis of porous materials for the very challenging capture of radioiodine.

## 3. Factors Affecting the Molecular Iodine Capture Performance of COFs

Research shows that the textural properties, primarily the surface area, pore size, and pore volume, of the adsorbent have crucial influences on the total I_2_ uptake capacity. However, another view holds that the affinity between the binding site and the I_2_ molecule, and the density of the binding sites, rather than the textural properties of the adsorbent play a decisive role in the I_2_ adsorption capacity. Many studies on COF-based iodine capture focus on exploiting the large surface areas and high porosity of porous adsorbents and regulating their binding sites.

### 3.1. Structural Characteristics of COFs

#### 3.1.1. Porosity

According to Jiang’s work [53], the pore volume is a key parameter in determining uptake capacity. They designed a series of 2D COFs with large surface areas and 1D open channels. These channels possessed various topologies from hexagonal to tetragonal and trigonal, and were free of specific binding sites and interchannel interpenetration. So, the possibility of a charge transfer and reaction were excluded. The channels could only uptake iodine in a physical adsorption mode driven by van der Waals forces. They proved that I_2_ capture does not require specific functionalization of the porous skeleton, and that pores of any shape or size can be 100% occupied by physical adsorption. However, a number of recent studies have cast doubt on this view. In addition, Yan’s group synthesized two kinds of thiophene-based COFs, PB-TT COF and PA-TT COF, with different morphologies using thieno[3,2-b]thiophene-2,5-dicarbaldehyde (A6) as the aldehyde monomer and tri(4-aminophenyl)benzene (M11) or tris(4-aminophenyl)amine (M8) as the amino monomer [48]. Among them, the PB-TT COF exhibited high iodine uptake up to 5.97 g·g^−1^ in vapor, which was attributed to its high specific surface area (1305.3 m^2^·g^−1^) and affinity binding sites (N and S) in the skeleton. The adsorption mechanism revealed that iodine was captured through a combined process of physisorption and chemisorption. Furthermore, An et al. offered four new COFs with various porosities, varying from microporous to mesoporous, via rationally turning COF linkages [52]. They found that Meso-COF-4 showed a larger pore size of 4.7 nm, but a lower iodine uptake of 3.3 g·g^−1^. That is, the iodine uptake capacities of COFs were not only determined by their pore volumes but also significantly related to their intrinsic pore sizes through the separation experiments. COFs with large pore sizes, pore volumes, or surface areas do not mean high iodine adsorption. Some COFs with different pore sizes, pore volumes, and surface areas for iodine capture are listed in Table 1. Focusing on the balance between the surface area, porosity, and adsorption position of the COF frame, and considering the comprehensive and special investigation, COF materials with excellent iodine capture performance are likely to be screened.

#### 3.1.2. π-Conjugated Systems

**Figure 3:**
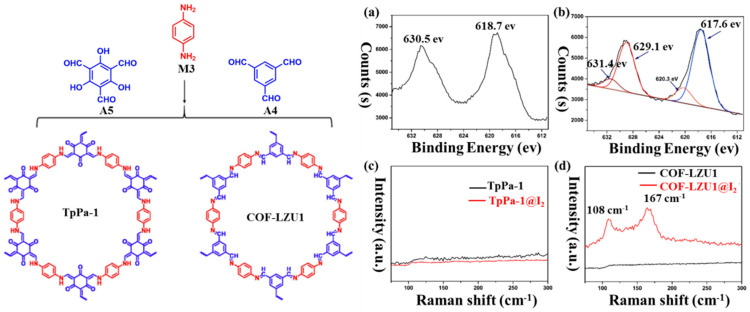
Left: schematic description of the synthesis of TpPa-1 and COF-LZU1. Right: XPS spectra of I_2_@TpPa-1 (**a**) and I_2_@COF-LZU1 (**b**), and Raman spectra of TpPa-1 (**c**) and COF-LZU1 (**d**) before and after volatile iodine adsorption. Reprinted/adapted with permission from Ref. [57]. Copyright (2019), Elsevier.

The design and construction of large and intact π—π-conjugated systems could also be an effective way of obtaining COF materials with outstanding performance in iodine uptake. As a proof of concept, Zhou et al. discovered two highly π-conjugated COFs, TFPB-BPTA-COF (A20-M16) and TFPB-PyTTA-COF (A20-M17), by incorporating pyrene, which exhibits excellent iodine uptake values up to 5.62 g·g^−1^ [56]. Adsorption mechanism studies show that the interaction of iodine molecules with the imine bonds and pore channels of highly conjugated frameworks lead to a combination of physical and chemical adsorption in the I_2_ capture process. Our group selected two COFs [57]—COF-LZU1 (A4-M1) with a whole π—π-conjugated structure, and TpPa-1(A5-M1) with a combination of π—π- and p-π-conjugated structures (Figure 3). Compared with TpPa-1, COF-LZU1 exhibits a much higher capacity for iodine enrichment. The XPS and Raman spectroscopy studies confirmed that the adsorbed iodine species in TpPa-1 existed as I_2_ and the process was physical adsorption driven by van der Waals forces, while the adsorption mode of COF-LZU1 was a combination of physical adsorption and chemisorption.

#### 3.1.3. Dimensions of COFs

As is known, the dimension and topological structure of COFs have important effects on their potential applications. Compared with 3D COFs, 2D COFs have lower costs, richer monomer species and more designable and adjustable structures. Therefore, current studies on iodine enrichment are mainly focused on 2D COFs, with emphases on improving the adsorption capacity of iodine through structural design, and on exploring the influence of structure on iodine adsorption performance.

Wang’s group demonstrated three ultra-stable -C-N- linked COFs—COF-TpgDB (A5-M1), COF-TpgBD (A5-M7), and COF-TpgTd (A5-M5)—via a straightforward synthesis method at ambient temperature [58]. The high-symmetry framework and large specific surface area of all three COFs permitted iodine to diffuse through, fulfill, and become trapped in all of the cavities. Each layer of the three COFs had multiple adsorption sites —C=O, -NH, and Ph—to attract the iodine species, leading to significantly large adsorption energies. Among them, COF-TpgDB had the lowest adsorption energy of 151.74 kcal/mol and the highest iodine adsorption capacity. Additionally, the adsorption mechanism was thoroughly studied via density functional theory (DFT).

**Figure 4:**
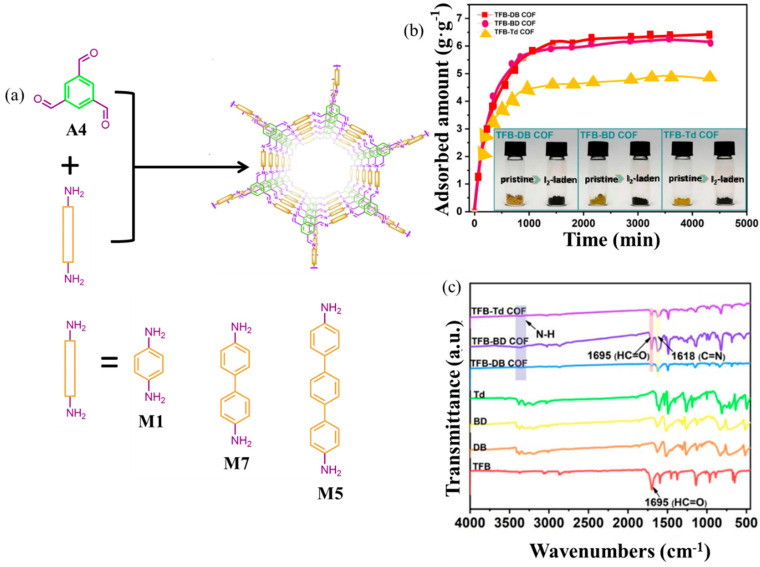
(**a**) Synthesis of TFB-DB, TFB-BD, and TFB-Td COFs. (**b**) IR spectra of the TFB, DB, BD, Td, and TFB-DB COFs, the TFB—BD COF, and the TFB-Td COF. (**c**) Adsorption curves of the investigated COFs. Reprinted/adapted with permission from Ref. [59]. Copyright (2021), American Chemical Society.

Subsequently, Song et.al [59] utilized three COFs—TFB-DB COF (A4-M1), TFB-BD COF (A4-M7), and TFB-Td COF (A4-M5)—with similar topologies to the COFs reported by Wang’s group (Figure 4), to carry out an experiment complementary to Jiang’s theory. The crystal structures and morphologies of the three candidate COFs were resolved via powder X-ray diffraction, FTIR spectra, solid-state ^13^C NMR spectroscopy, scanning electron microscopy, and transmission electron microscopy. Although the only difference between these three COFs was the linker, the binding energy was obviously different. After 54 h, the maximum adsorption capacities of iodine reached 6.40 g·g^−1^, 6.23 g·g^−1^, and 4.97 g·g^−1^ for TFB-DB COF, TFB-BD COF, and TFB-Td COF, respectively. The research confirmed that not only would the pore size and pore volume remarkably affect iodine uptake, but so would the binding energy.

However, the iodine adsorption rate of most 2D COFs is very slow, which is a problem that cannot be ignored. As shown in Table 1, it usually takes dozens of hours to reach equilibrium under typical iodine adsorption conditions. Therefore, determining how to reasonably design the structure of 2D COFs to effectively improve their adsorption rate for iodine while ensuring their adsorption capacity is still one of the key issues to be solved.

**Figure 5:**
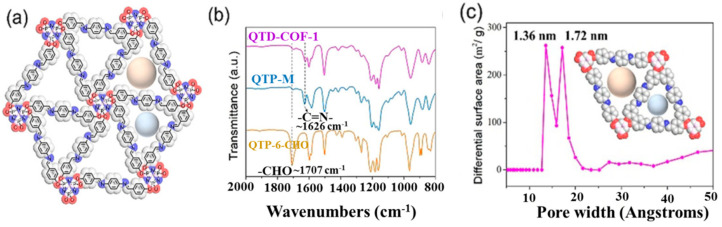
(**a**) Crystal structure of QTD-COF-1. (**b**) FT-IR spectra. (**c**) Pore-size distribution for QTD-COF-1.Reprinted/adapted with permission from Ref. [60]. Copyright (2020), John Wiley and Sons.

The iodine adsorption rates of 3D COFs are somewhat better than those of traditional 2D COFs. 3D COFs have open spatial structures and multidirectional and interconnected pore properties, which enable better permeability. The 3D pore characteristics are conducive to the diffusion of the target adsorbate in the structure, accelerating the mass transfer rate and shortening the equilibrium time. Guo et.al reported a unique “Q-3D” (quasi-three-dimensional) COF [60], namely QTD-COF-1 (A16-M1), which was prepared via self-assembly polymerization under solvothermal conditions, with CTP-6-CHO (hexa(4-formyl-phenoxy)cyclotri-phosphazene, A16) as the node and p-phenylenediamine as the linker (Figure 5). The structure of QTD-COF-1 between two and three dimensions shows abundant stereoscopic lateral channels. This unique pore structure enables the guest molecules to enter the framework of the QTD-COF-1 through both the frontal and the lateral pathways, thus giving it an excellent iodine adsorption capacity of 4.62 g·g^−1^. Additionally, the adsorption capacity of QTD-COF-1 reached 80% of the saturated adsorption capacity within just 3 h.

**Figure 6:**
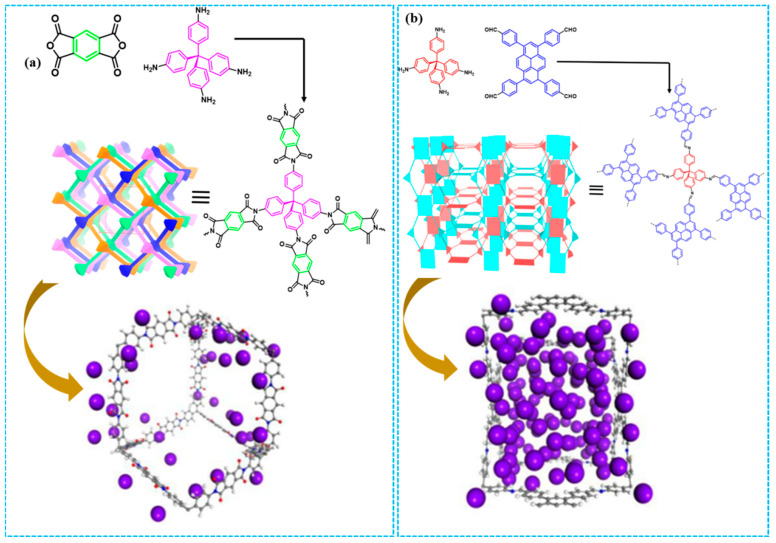
(**a**) Schematic representation of the synthesis and extended structure of PI-COF-5, and I_2_ adsorption configurations in PI-COF-5. Reprinted/adapted with permission from Ref. [62]. Copyright (2015) American Chemical Society. (**b**) Schematic representation of the synthesis and extended structure of 3D-Py-COF, and I_2_ adsorption configurations in the structure. Reprinted/adapted with permission from Ref. [63]. Copyright (2016) American Chemical Society.

Wang et al. identified a 3D COF, TTF-TAPT-COF (A16-M9), by introducing tetrathiafulvalene functional groups [49]. The TTF-TAPT-COF exhibited a superior iodine vapor adsorption capacity of 5.02 g·g^−1^ at 348 K and ambient pressure with an adsorption kinetics value of 0.515 g/(g·h). Lan and co-workers screened the best existing COF for I_2_ capture (3D-Py-COF, 16.7g·g^−1^) through a large-scale computational screening study of 187 COFs [61]. Additionally, they designed a new 3D COF (3D-Py-COF-TANM) with an even higher I_2_ uptake of 19.9 g·g^−1^ on this basis. However, not all the 3D-COFs presented excellent adsorption performance for I_2_. For example, PI-COF-5 (A22-M12) [62] has similar structural features (pore size, surface area, void fraction, and dia topology) to the best-performing COF, 3D-Py-COF (A20-M12) [63], but its I_2_ uptake is very low (Figure 6). In addition, although some 3D COFs show fast adsorption rates and excellent adsorption capacity, the limited available 3D monomers are complex and difficult to synthesize, and quite expensive to obtain commercially, resulting in a very limited number of 3D COFs. Other than that, the difficulty in the structural analysis of 3D COFs, especially those with interpenetration structures, has severely restricted their development in iodine capture application.

#### 3.1.4. Flexibility of the Skeleton

COFs bearing flexible monomers generally possess variable lattice sizes, which may endow them with unprecedented application values. In addition, the energy decomposition analysis suggests that the adsorption energies are mainly derived from the deformation energies of the COF. Thus, the increase in their flexible self-adaptive ability can significantly increase the adsorption rate and capacity of COFs to iodine.

**Figure 7:**
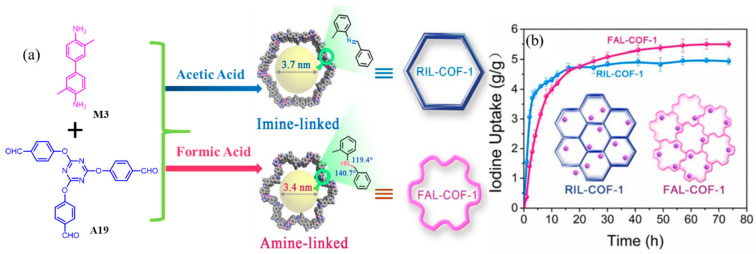
(**a**) Synthetic routes and simulated structures of FAL-COF-1 and RIL-COF-1. (**b**) Gravimetric iodine uptake of FAL-COF-1 and RIL-COF-1. Reprinted/adapted with permission from Ref. [64]. Copyright (2021), John Wiley and Sons.

Zhang et al. reported, for the first time, a series of flexible amine-linked COFs (A19-M3) with high crystallinity synthesized by formic acid, with its unique catalytic and reductive bifunctional properties, rather than acetic acid [64]. The BET surface area, pore volume and pore size of FAL-COF-1 are 168 m^2^·g^−1^, 0.32 cm^3^·g^−1^, and 3.4 nm (Figure 7a). Compared with rigid RIL-COF-1, the flexible structural framework of FAL-COF-1 does shrink the aperture of the material. Nevertheless, the iodine adsorption capacity of FAL-COF-1 is significantly increased (Figure 7b). Moreover, the flexible characteristics of FAL-COF-1 were demonstrated by studying the interactions between the host framework and the guest organic solvent molecules, such as ethyl alcohol, tetrahydrofuran, acetone, and acetonitrile. The results show that the pore and channel of FAL-COF-1 have a certain elasticity and self-adaptability.

**Figure 8:**
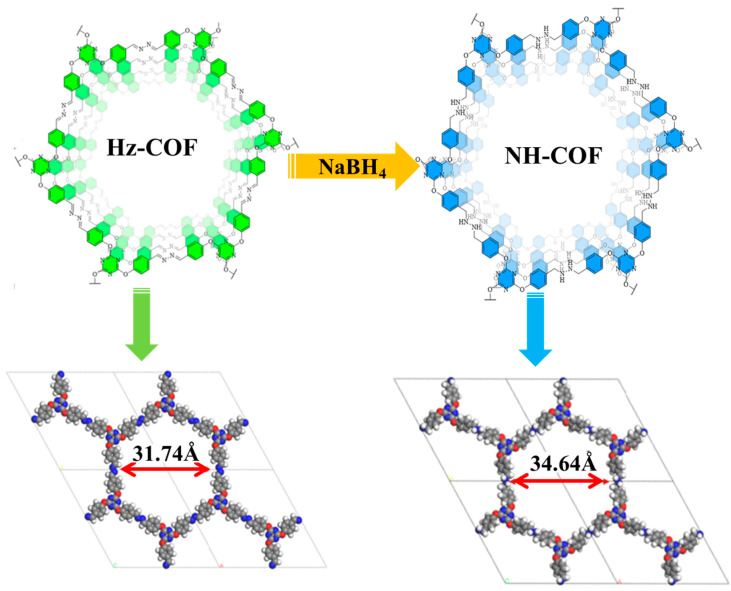
An illustration of Hz-COF and NH-COF, and their corresponding simulated views. Reprinted/adapted with permission from Ref. [65]. Copyright (2022), Elsevier.

Very recently, Dinari’s group compared the iodine adsorption performance of a flexible amine-linked COF (NH-COF) with that of a rigid imine-linked COF (Hz-COF) [65]. Hz-COF (A19-M21) was prepared traditionally using 2,4,6-tris(4-formyl phenoxy)-1,3,5-triazine and hydrazine, and then, subjected to reductive conditions to quantitatively reduce the hydrazone linkages to NH-COF (Figure 8). The NH-COF represents a 2.60 g·g^−1^ uptake compared to 2.05 g·g^−1^ for Hz-COF. It is considered that the flexibility of the NH-COF scaffold compared to the Hz-COF, and the formation of strong N-H···I between the NH moieties and I_3_- groups, are responsible for the superior iodine capture activity.

**Figure 9:**
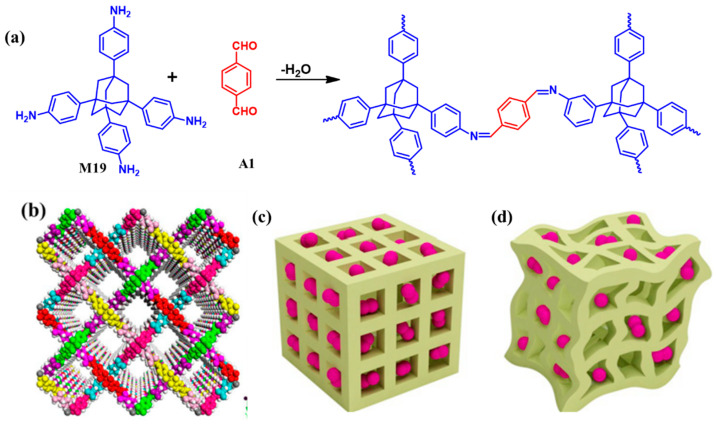
(**a**) Synthesis of COF-DL229. (**b**) Structural representations of eightfold interpenetrated **dia** network of 3D COF-DL229. Schematic representation of plausible iodine adsorption behavior in the ordered COFs (**c**) and the deformed porous materials (**d**). Reprinted/adapted with permission from Ref. [67]. Copyright (2018), John Wiley and Sons.

Guo and co-workers constructed TPT-BD COF (A19-M7) and TPT-DHBD COF (A19-M20) based on the flexible module (2,4,6-tris(4-formylphe-noxy)-1,3,5-triazine, A19) through combinatorial copolymerization [66]. Among them, TPT-DHBD_X_ COFs (the X value is directly related to the proportion of DHBD containing -OH groups, X = 0, 50, 100) which are rich in nitrogen and contain abundant aromatic rings and C = N bonds, displayed iodine uptake of 4.77 g·g^−1^, 4.24 g/g, and 3.77 g·g^−1^, respectively. The mechanism of iodine enrichment indicated that iodine adsorption could occur simultaneously at the imine linkage, triazine ring, and phenyl ring of the materials. Wang and co-workers presented a “soft” 3D COF (COF-DL229, A1-M19), which was synthesized by 1,3,5,7-tetrakis(4-aminophenyl)-adamantane (TAPA) and 1,4-phthalaldehyde (PTA) under solvothermal conditions [67]. The phenyl linker endowed the edge parts with three phenyl groups linked by two imine bonds, leaving the skeletons with structural flexibility (Figure 9). COF-DL229 exhibited excellent performance in capturing iodine vapor (4.7 g·g^−1^). The state of the iodine inside the 1D channels of COF-DL229 were mainly I_3_^−^ and I_5_^−^ anions. Iodine adsorption causes prominent effects on the local pore wall structures to trigger structural deformation. PXRD measurements suggested that COF-DL229 remains in the framework structure without a change in the lattice, even upon the uptake of iodine. This 3D COF is capable of producing local structural deformations that prevent iodine from entering some spaces. However, this structural flexibility allows the COF samples to be reused many times.

Although flexible COFs have good iodine capture performance, the flexibility and variability of their building units in the three-dimensional direction make it relatively difficult to construct flexible structural frameworks. Additionally, the increased flexibility usually results in irregular polymerization and worse crystallinity of COFs.

### 3.2. Electron-Rich Functional Groups

Electron-rich groups, such as -NH-, -NH_2_, -C=N-, -OCH_3_, aromatic rings, tetrathiafulvalene, and heterocycles (S, P), can adsorb electron-deficient I_2_ by forming charge-transfer complexes, thereby increasing the adsorption capacity and adsorption rate by facilitating the modulation of the adsorption-driving force between iodine and functional group-modified COF absorbers. Different functional groups have different binding energies to iodine. Even for the same functional groups, for example, -C=N-, when they link to different linkers, the iodine binding energies have differences. To prepare electron-rich adsorbents, various strategies have been developed, including the incorporation of ionic sites or heterocyclic moieties, the construction of π-conjugated networks, doping with heteroatoms, and combinations thereof.

**Figure 10:**
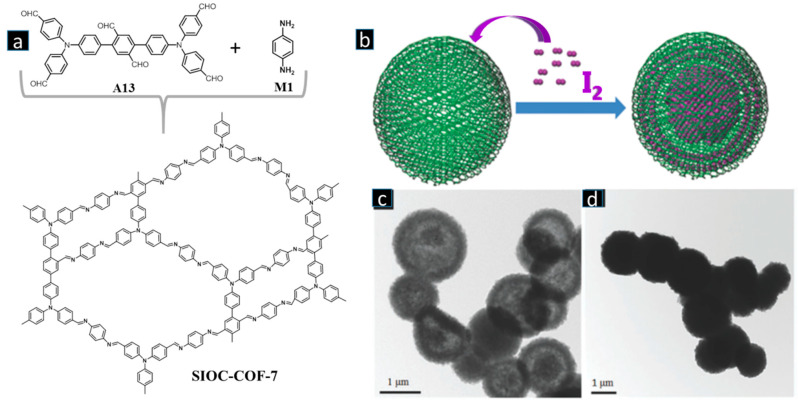
(**a**) Synthesis and structure of the heteropore COF SIOC-COF-7. (**b**) Cartoon illustration of iodine capture in the spherical COF. TEM images of SIOC-COF-7 (**c**) before and (**d**) after the COF was exposed to iodine vapor. Reprinted/adapted with permission from Ref. [47]. Copyright (2017), Royal Society of Chemistry.

Among the vast COFs reported thus far, imine-bonded architectures occupy the largest proportion owing to the facile availability of the multialdehyde and multiamine precursors. Zhao’s group reported a hollow spherical heteropore COF (SIOC-COF-7, A13-M1) [47], which combined the features of highly ordered internal structures and hierarchical porosity. The two main pore-size distributions were 5.0 and 11.8 Å, respectively. SIOC-COF-7 was synthesized via the condensation of 4,4″-bis(bis(4-formylphenyl)amino)-[1,1′:4′,1″-terphenyl]-2′,5′-dicarbaldehyde (A13) and 1,4-diaminobenzene (M1). This is the first COF-base iodine adsorbent, for which an ultrahigh iodine uptake of 4.81 g·g^−1^ was obtained after 48 h. The authors envisioned that the inner cavities of the COF microspheres, as well as their porous spherical shells, should be responsible for iodine capture (Figure 10). However, the detailed adsorption mechanism was not studied in their work. We believe that in addition to the well-ordered network of the hetero-COF, the abundant aromatic rings and high nitrogen content should also be favorable for iodine enrichment. Li’s group employed a stable N-containing COF (TAPA-PDA COF, A1-M8) for efficient and reversible volatile iodine capture (5.09 g·g^−1^) [68]. It is considered that the large surface area (685 m^2^·g^−1^) and ordered channel distribution of TAPA-PDA COF enabled the even dispersion of a high density of N-chelating sites throughout the channel surface of the TAPA-PDA COF, which was conducive to iodine capture.

**Figure 11:**
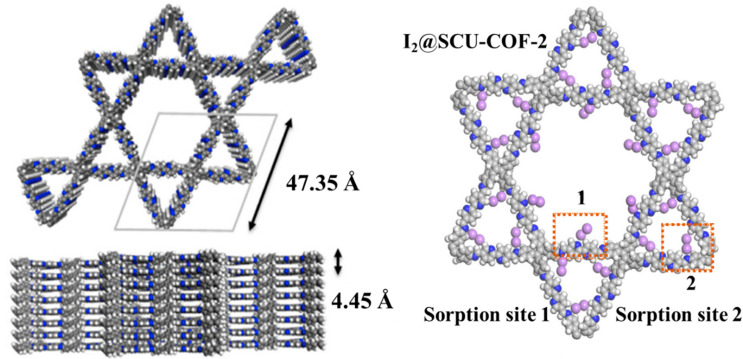
**Left**: top view and side view of SCU-COF-2. **Right**: adsorption of I_2_ on SCU-COF-2. Reprinted/adapted with permission from Ref. [69]. Copyright (2021), Elsevier.

From the above works, it can be found that although these COFs rich in C=N groups show good iodine capture capacity, they designed studies show them to have fewer chemically active sites. In view of this, many researchers focus on increasing the density of active sites in COFs. Wang and co-workers designed and synthesized a robust N-rich COF (SCU-COF-2, A14-M18) by introducing a bipyridine group into the building blocks for the simultaneous capture of both iodine gas and methyl iodide. The monomer 2,2′-bipyridyl-5,5′-dialdehyde (A14) formed stable charge-transfer complexes with the π electron-rich aromatic framework, and multiple N atoms interacted with iodine [69]. The uptake capacity for iodine by SCU-COF-2 reached as high as 6.0 g·g^−1^ at 96 h, which is beneficial for the introduction of dense pyridine N atoms, providing a stronger affinity for iodine molecules (Figure 11). DFT calculations revealed that both I_2_ and CH_3_I molecules prefer to occupy the sites near the N atoms of pyridine rings in the hexagonal hollow and at the intersection angle sites in the triangular hollow.

**Figure 12:**
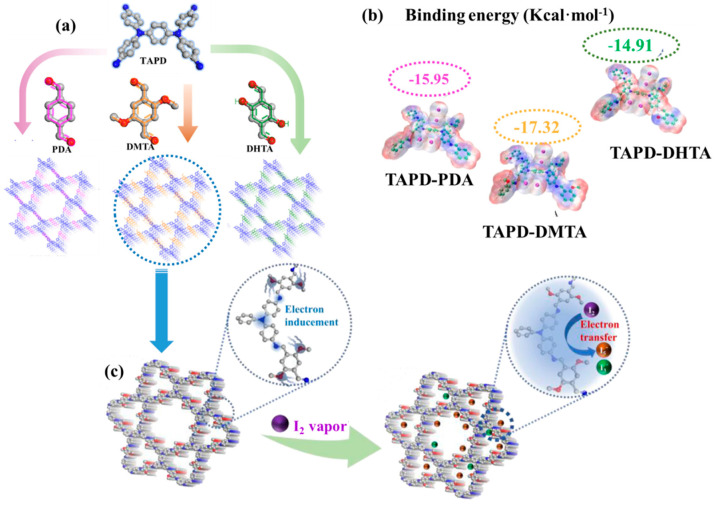
(**a**) Schematic diagram of the COF synthesis. (**b**) Schematic illustration of the iodine uptake mechanism of TAPD-DMTA COF. (**c**) The binding energy with iodine and ESP analysis. Reprinted/adapted with permission from Ref. [70]. Copyright (2022), Elsevier.

Very recently, Jiang and co-workers designed and constructed three 2D COFs from a spatially twisted four-link multi-nitrogen node building block (tetrakis(4-aminophenyl)-1,4-benzenediamine, M13) [70]. At the same time, different electron-donating groups were introduced into COFs to further enhance the adsorption affinity of the active sites for iodine (Figure 12). Thus, the obtained structures not only display a high density of active adsorption sites that can fully guarantee the iodine adsorption capacity, but also exhibit space adaptation because the hexagonal and triangular pore characteristics of the TAPD-PDA COF (A1-M13), TAPD-DMTA COF (A3-M13), and TAPD-DHTA COF (A2-M13) all have high crystallinity. The BET surface areas are 194.1, 415.2, and 213.6 m^2^·g^−1^, respectively. All of them have two different pore sizes and their average pore sizes are 1.81/2.78 nm, 1.23/2.60 nm, and 1.56/2.73 nm, respectively. All three COFs are in AA stacking mode and a Kagome topological configuration. The maximum adsorption capacities of TAPD-PDA COF, TAPD-DMTA COF, and TAPD-DHTA COF are 5.09, 5.54, and 4.02 g·g^−1^, respectively. The iodine capture mechanism research indicates that the iodine molecules are basically converted into poly-anion iodides, and thus, are accompanied by chemical adsorption process. Jiang’s research shows that the introduction of -OCH_3_ into the skeleton can increase the electron cloud density around the N active site through a charge-induced effect, thereby enhancing the binding energy between COFs and iodine, and thus, achieving a higher iodine adsorption capacity. However, the introduction of -OH tends to form intramolecular hydrogen bonds with the N of the ortho-imine, which leads to a decrease in electron density around the active sites, and finally, results in a decrease in iodine adsorption capacity.

Pan et al. identified a novel COF, BTT-TAPT-COF (A11-M9), through the S-rich monomer benzo[1,2-b:3,4-b′:5,6-b″]trithiophene-2,5,8-tricarbaldehyde (A11) and N-rich monomer 1,3,5-tris-(4-aminophenyl)triazine (M9) [71]. BTT-TAPT-COF contains abundant S and N active sites and extended π conjugation with inherent microporosity, and they found that it could adsorb I_2_ reversibly with a capacity of 2.76 g·g^−1^. In particular, the captured iodine in the BTT-TAPT-COF could be easily released from the COF by immersing the I_2_@BTT-TAPT-COF in methanol at room temperature, and the COF still retained more than 99% of its initial iodine uptake capacity after five cycles. Nevertheless, although BTT-TAPT-COF has a porous character, abundant S and N active sites, as well as a large π-conjugated network structure, its iodine adsorption capacity is not excellent. The reason may be that the presence of the triazine ring units leads to a decrease in the affinity between the active site (C=N) and iodine.

Moreover, Jiang’s group identified two N-rich 2D COFs, USTB-1 (A23-M7) and USTB-2 (A23-M14), which were transformed by BPPOC with a single-crystal X-ray diffraction structure [72]. The cage-derived COFs exhibited iodine vapor adsorption capability with the uptake of 5.80 g·g^−1^ for USTB-1c. The mechanism investigation unveiled the superiority of N atoms over S atoms for POCs in iodine vapor capture with the assistance of definite crystal structures. The results of the DFT calculations suggested a stronger binding interaction with the I_3_^−^ and I_5_^−^ of the former 2,2′-bipyridine moiety due to its N-rich moiety. At the same time, the work of Song’s group also proved that the binding energy would remarkably affect iodine capture [58]. They employed a series of different COFs, and there was at least one iodine capture site corresponding to their building blocks for each COF. Each potential binding site was separately calculated. For BTT-TAPT (A11-M9), the calculation results showed that the binding energies of I_3_^−^ ions at the potential sites of -S-, -N-, and the conjugate sites were −31.23, −11.35, and −10.38 kcal·mol^−1^, respectively, which indicated that it was hard for I_3_^−^ to bind at the -S- site. Therefore, the -S- site was not a good iodine adsorption site, which is consistent with Jiang’s conclusion.

Additionally, Ke’s group reported a phosphine-based COF (P-COF, A9-M7) with abundant heteroatom (P, N) adsorption sites, a large surface area (1056 m^2^·g^−1^), and a large π-conjugated skeleton [73]. This P-COF exhibited exceptional iodine uptake in both volatile iodine and iodine/cyclohexane solution with adsorption capacities of 6.19 g·g^−1^ and 1.30 g·g^−1^, respectively, suggesting that P-COF may be a potential candidate for radioiodine capture. FT-IR and XPS spectra indicate that iodine adsorption could occur simultaneously at the aromatic ring and C=N linkage. However, the mechanism of sorption sites in the adsorption process has not been revealed, and no PXRD diffraction peaks can be observed after the first recovery of P-COF.

**Figure 13:**
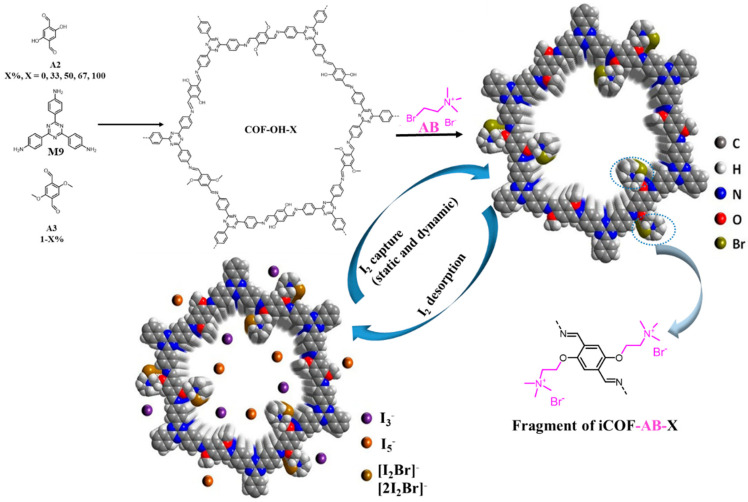
Synthetic procedure of multivariate COF-OH-X and iCOF-AB-X materials. Schematic illustration of iCOF-AB-X for I_2_ capture. Reprinted/adapted with permission from Ref. [51]. Copyright (2022), John Wiley and Sons.

Another notable strategy effectively promoting I_2_ adsorption is the generation of cationic sites in the adsorbent, for instance, guanidine, tetrathiafulvale, and (2-bromoethyl)trimethylammonium bromide, which can bind polyiodide anions via Coulomb interactions. Recently, Han’s group identified a series of ionic COFs (iCOFs) via the “multivariate” synthetic strategy combined with post-synthesis modification [51]. The so called “multivariate” strategy is based on the use of two or more structurally homologous monomers to simultaneously incorporate multiple groups into the COF structure. The initial COF was constructed from 2,4,6-tri(4-aminophenyl)-1,3,5-triazine (M9) and a mixture of 2,5-dihydroxyterephthalaldehyde (A2) and 2,5-dimethoxyterephthalaldehyde (A3). The density of the -OH groups in the COF (COF-OH-X, where X represents the molar percentage of DHTA relative to the total of A2 and A3) was tuned by varying the ratio of A2 to A3 in the synthetic precursor. All COF-OH-X exhibit high crystallinity and large initial surface areas, which ensure that significant porosity can be retained after ionic modification, and have high densities of imine and triazine moieties throughout their entire framework. Then, the -OH groups were utilized as reactive sites to modify the COF-OH-X with (2-bromoethyl)trimethylammonium bromide (AB), resulting in iCOF-AB-X (Figure 13). iCOF-AB-50 was determined to be the best-performing material though a trade-off between the number of binding sites and textural properties. COF-OH-50 and iCOF-AB-50 were employed as examples to compare research on iodine capture performance. The I_2_ adsorption capacities of COF-OH-50 and iCOF-AB-50 under static conditions were 6.49 and 10.21 g·g^−1^, respectively. Additionally, the I_2_ adsorption capacities of COF-OH-50 and iCOF-AB-50 under dynamic conditions and at room temperature were 1.70 g·g^−1^ and 2.79 g·g^−1^, respectively. In particular, compared with other reported COF-based adsorbents, iCOF-AB-50 exhibited the highest I_2_ adsorption capacity under both static and dynamic conditions. Additionally, the authors also investigated the I_2_ capture performance in the presence of water. This groundbreaking work provided a new design strategy for the development of high-performance iodine adsorption materials.

**Figure 14:**
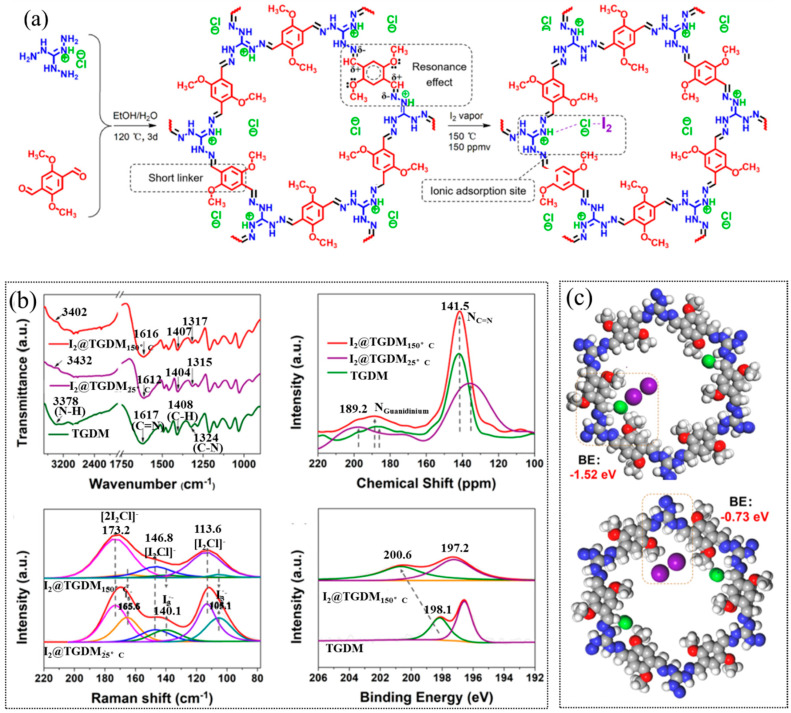
(**a**) Schematic illustration of the design and synthesis of TGDM and explanation of its high stability and I_2_ uptake mechanism. (**b**) FT-IR spectra, solid-state ^15^N NMR spectra, Raman spectra, and XPS Cl 2p spectra of TGDM and iodine-loaded samples. (**c**) Two typical configurations of I_2_ interacting with the guanidinium group in TGDM and the corresponding calculated binding energies. Reprinted/adapted with permission from Ref. [74]. Copyright (2022), American Chemical Society.

Subsequently, they reported a novel stable guanidinium-based COF (TGDM) through the reaction of thiamine-guanidine hydrochloride (TG_Cl_) and 2,5-dimethoxyterephthalaldehyde (A3), which can efficiently capture I_2_ under industrial conditions [74]. Ionic guanidine groups provide strong adsorption sites at 150 °C and 150 ppmv of I_2_ through coulombic interactions to trap I_2_. The saturated I_2_ uptake capacity of TGDM was determined to be 29.24 wt%, which is higher than that of the well-known iCOF-AB-50 (8.27 wt%). This may be due to the different ionic sites (quaternary ammonium groups for iCOF-AB-50), and also the fact that the ionic site density of iCOF-AB-50 is lower than that of TGDM (number of ionic sites per gram: 1.09 × 10^21^ vs. 1.59 × 10^21^). Remarkably, TGDM can be easily recycled and reused without losing its adsorption capacity. The characterization and theoretical calculations indicate that iodine adsorption follows different mechanisms at low and high temperatures (Figure 14). Additionally, among the multiple types of adsorption sites in TGDM (such as imine and benzene rings), only the ionic guanidinium groups can bond to I_2_ through strong Coulomb interactions under harsh conditions. Therefore, we can speculate that the number of strong ionic binding sites in COF, rather than structural features, is decisive of I_2_ adsorption capacity under harsh conditions. Han’s group produced excellent work in this regard. Their strategies, elaborate design of monomers, and “multivariate” synthetic strategy could stimulate the development of new adsorbents for high I_2_ capture performance.

**Figure 15:**
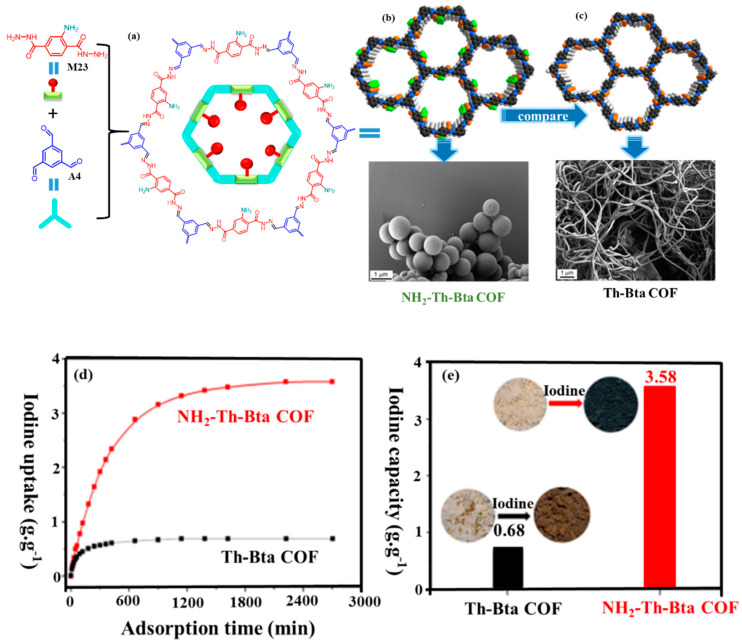
(**a**) Synthesis of the amine-functionalized NH_2_-Th-Bta COF using the site-selective synthetic strategy. (**b**) Simulated structure and SEM image of Th-Bta COF. (**c**) Simulated structure and SEM image of NH_2_-Th-Bta COF. (**d**) Iodine vapor adsorption on Th-Bta and NH_2_-Th-Bta COFs. (**e**) Comparison of the iodine uptake capacities of Th-Bta and NH_2_-Th-Bta COFs. Reprinted with permission from [75]. Copyright (2021), American Chemical Society.

Recently, Zhang et al. developed a site-selective synthetic strategy for the facile preparation of an amine-functionalized hydrazone-linked COF (NH_2_-Th-Bta COF, A4-M23) for the first time [75]. A brilliant monomer 2-aminoterephthalohydrazide (NH_2_-Th) bearing both amine and hydrazide functionalities was designed to react with benzene-1,3,5-tricarbaldehyde (Bta). The different activities of the amine and hydrazide groups toward aldehyde underpinned the synthesis of the unprecedented NH_2_-Th-Bta COF via a simple and straightforward route (Figure 15). The nonfunctionalized Th-Bta COF (A4-M22) counterpart was prepared as a control sample. The FT-IR spectra of Th-Bta and NH_2_-Th-Bta COFs indicate the occurrence of the Schiff-base reaction. The SEM images of the two are obviously different: the image for Th-Beta shows a well-defined nanofibrous morphology, while the image for the NH_2_-Th-Bta COF presents a relatively uniform spherical morphology. The NH_2_-Th-Bta COF with abundant free amine groups decorated in the periodic 1D channel exhibited a dramatically enhanced iodine uptake capacity (3.58 g·g^−1^) in comparison to that of the nonfunctionalized Th-Bta COF counterpart (0.68 g·g^−1^), despite its specific low surface area (10 m^2^·g^−1^). This work proved that the introduced free amine group plays an essential role in enhancing iodine adsorption capacity, which gives rise to a charge-transfer complex with the iodine molecule. Moreover, the NH_2_-Th-Bta COF possessed exceptional cycling capability and retained high iodine uptake, even after six cycles. This strategy of increasing the density of active sites improves the iodine adsorption capacity, which sheds light on the future design of advanced porous materials. It is reasonable to speculate that if such a strategy can be used to obtain COFs with large enough surface areas, the iodine adsorption capacity will inevitably increase significantly.

**Figure 16:**
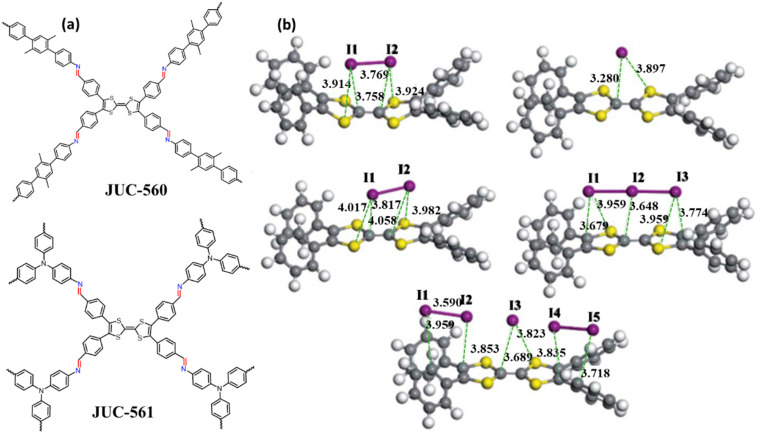
(**a**) Building unit of JUC-560 and JUC-561. (**b**) Optimized geometries of TTF·^+^ and polyiodides. Reprinted/adapted with permission from Ref. [50]. Copyright (2021), Royal Society of Chemistry.

Furthermore, tetrathiafulvalene-based (TTF) COFs have attracted much attention for their excellent iodine adsorption performance. According to the work of Fang’s group [49]. The reason that COFs containing tetrathiafulvalene functional groups exhibit both ultrahigh iodine adsorption capacity and ultrafast adsorption kinetics is that there is strong chemisorption between polyiodides and TTF·^+^. Mechanistic studies have shown that TTF units in these COFs are subjected to a charge-transfer from TTF to iodine, forming oxidized TTF·^+^ radical cations and poly-iodides, which, at last, are tightly bound to the cationic framework through electrostatic interactions (Figure 16).

In principle, COFs can be functionalized with the desired adsorption ionic sites through the design of appropriate monomers or post-synthesis modification; through this, they can achieve a high I_2_ adsorption capacity and adsorption rate due to their strong Coulomb interactions between ionic sites and polyiodide anions. However, in practice, it is very challenging to incorporate ionic sites into COFs while maintaining high crystallinity and accessible porosity [55,76]. In addition to the above internal factors, some external factors, such as adsorption temperature, I_2_ concentration, pressure, static/dynamic conditions, and other different test conditions, will also significantly affect the iodine adsorption performance of COFs.

**Table 1:** Some structural features and adsorption properties of volatile I_2_ (348~350 K and 1 bar) of the reported COFs.

COFs	BET (m_2_·g^−1^)	Pore Size (nm)	Pore Volume (cm_3_·g^−1^)	I_2_ Uptake (g·g^−1^)	Adsorption Equilibrium Time (h)	Adsorption Mechanism *^a^*	Ref.
PA-TT COF	48.6	2.45	0.112	5.1	12	P-C	[48]
PB-TT COF	1305.3	3.67	0.986	5.97	72	P-C	[48]
TTF-TAPT	461	/	0.28	5.02	18	P-C	[49]
JUC-560	1815	2.6	1.11	5.2	14	P-C	[50]
JUC-561	2359	2.55	1.92	8.19	18	P-C	[50]
TPB-DMTP	1927	3.3	1.28	6.26	48	P	[53]
TTA-TTB	1733	2.2	1.01	4.95	96	P-C	[53]
Micro-COF-1	816	1.6	0.59	2.9	75	P-C	[54]
Micro-COF-2	1056	1.7	0.71	3.5	75	P-C	[54]
Meso-COF-3	982	4.0	0.84	4	75	P-C	[54]
Meso-COF-4	926	4.7	1.01	3.3	75	P-C	[54]
COF-TpgDB	209.6	6.8	0.36	2.75	65	P-C	[58]
COF-TpgBD	217.9	8.3	0.46	1.81	65	P-C	[58]
QTD-COF-1	/	1.36/1.72	/	6.29	20	P-C	[60]
NH_2_-Th-Bta	10	2.4	/	3.58	37	P-C	[75]
Th-Bta	22	2.6	/	0.68	37	P-C	[75]
TJNU-201	2510	1.4	/	5.625	96	P-C	[77]
TJNU-202	714	1.7	/	4.82	96	P-C	[77]
TJNU-203	1833	0.98	/	5.885	100	P-C	[78]
TJNU-204	2048	0.89	/	5.335	100	P-C	[78]
TPT-Azine	1020	2.5	0.65	2.19	/	P	[79]

*^a^* C—chemical; P—physical.

In short, I_2_ adsorption capacity is determined by the collective effects of multiple factors: the textural properties (surface area, pore size, and pore volume) of the adsorbent, the affinity between the binding site and the I_2_ molecule, and the density of binding sites. Therefore, an optimal balance between these factors is essential for achieving high adsorption capacity. Based on the above theory, knowledge of how to construct COFs with regular channels and improve the density of adsorption sites on the materials—while effectively ensuring high activity and efficiency of these adsorption sites, as well as high affinity for iodine—has become the key to preparing high-performance iodine capture materials.

## 4. Conclusions and Outlook

As a sustainable and low-carbon energy supply, nuclear energy is expected to play a more important role in the future. Radioactive iodine in the waste of the nuclear industry is one of the important factors hindering its popularization. COFs exhibit unique properties including large surface areas, ultrahigh porosity, tunable pore sizes, and relatively high chemical/thermal stability combined with tailorable architecture, which are not seen in other porous materials. Because of these appealing properties, vast research has been conducted on COF-based materials for vapor iodine capture in recent years.

In general, an outstanding COF-based material for radioiodine capture that can be used in industry should satisfy the following requirements: (I) high adsorption capacity and adsorption kinetics for vapor iodine under industrial conditions; (II) high selectivity within a mixture of competitive gases; (III) long retention time of the adsorbed iodine; (IV) and excellent recyclability and low-cost.

According to recently published works, there is still much room for further improvement in the iodine capture performance of COF materials, which also provides great opportunities for researchers in this field to: (I) pay attention to the selective adsorption of COFs on vapor iodine under industrial conditions; (II) develop high-performance adsorbent COFs that can efficiently capture low-concentration I_2_ at high temperatures and under other harsh industrial conditions; (III) design and synthesize 3D COFs; and (IV) evaluate the I_2_ adsorption capacities of COFs under dynamic conditions. These strategies may yet lead to progress in the construction of COFs with enhanced adsorption capacity and adsorption kinetics under industrial conditions.

In summary, we provide an overview of the research progress in the iodine capture application of COF materials, which is a very active subject. We hope this work will provide guidance for the design and synthesis of COFs with excellent iodine capture performance, and inspire innovations in this emerging field. Additionally, we believe that in the near future, this research area will experience rapid growth followed by industrial application.

## Data Availability

Not applicable.
